# Computational Approaches for Discovering Virulence Factors in *Coccidioides*

**DOI:** 10.3390/jof11100754

**Published:** 2025-10-21

**Authors:** Arianna D. Daniel, Vikram Senthil, Katrina K. Hoyer

**Affiliations:** 1 Quantitative Systems Biology Graduate Program, University of California Merced, Merced, CA 95343, USA; 2 Department of Molecular and Cell Biology, School of Natural Sciences, University of California Merced, Merced, CA 95343, USA; vsenthil3@ucmerced.edu; 3 Health Sciences Research Institute, University of California Merced, Merced, CA 95343, USA

**Keywords:** computational biology, virulence factors, respiratory dimorphic fungi, *Coccidioides*, fungal pathogenesis, bioinformatics, machine learning

## Abstract

Emerging respiratory dimorphic fungi, including *Coccidioides*, pose a growing public health threat due to their ability to cause severe disease and the limited therapeutic options. A growing gap exists between rapidly expanding computational data and slower traditional experimental methods for virulence factor identification, limiting progress in fungal pathogenesis research and therapeutic development. This review presents a framework for integrating computational and experimental methodologies to accelerate virulence discovery in *Coccidioides*. We examine predictive tools for adhesins, transporters, secreted effectors, carbohydrate-active enzymes (CAZymes), and secondary metabolites, plus therapeutic target prioritization strategies based on druggability, selectivity, essentiality, and precedent. Examples from *Coccidioides* and other World Health Organization-designated emerging fungi highlight how computational pipelines clarify pathogenic mechanisms and guide experimental design. We also assess machine learning, structural prediction, and reverse vaccinology approaches for enhance target discovery. By applying computational advances to *Coccidioides* research with experimental validation, this integrated approach can guide future antifungal drug and vaccine development.

## 1. Introduction

The fungal kingdom contains roughly 3.5 to 5.1 million species, of which about 200–300 species cause disease in humans [1]. Most opportunistic fungi cause infections only in immunocompromised individuals, but a number can also infect apparently healthy people who may have undiagnosed underlying immune defects that become apparent only when they cannot clear certain fungal infections. In 2022, the World Health Organization (WHO) reported a collection of significant emerging pathogens to guide research efforts and policies, raise global awareness, and improve clinical management of fungal infections [2]. Respiratory pathogenic fungi maintain two lifecycles, shifting between a filamentous (mycelia) form and a yeast-like form, a crucial mechanism for virulence and adaptation to host environments [3]. Understanding these mechanisms is crucial for combating the growing threat from respiratory fungal pathogens like *Coccidioides.* This knowledge exposes gaps in our understanding of virulence mechanisms, especially as these organisms adapt to changing environments and develop resistance to current medications [4]. Traditional experimental approaches target and explore pathway-specific processes, but as fungal genomes are sequenced faster than their proteins can be characterized, these methods struggle to keep pace. This complexity creates a bottleneck between data generation and biological relevance. The challenge is particularly acute for understudied pathogens like *Coccidioides*, where limited resources and biosafety constraints make large-scale functional studies difficult despite the clinical need for better treatments.

Conventional methods for studying virulence factors move slowly and depend on costly, specialized equipment. The combination of advanced genomic technologies, powerful computational tools, and machine learning creates opportunities to overcome these roadblocks. Computational biology offers/provides robust tools to analyze fungal proteomes, predict virulence factors with greater accuracy, and identify promising targets for laboratory investigation. These approaches bridge the gap between generating massive datasets and conducting targeted experimental study. *Coccidioides immitis* and *C. posadasii* cause systemic mycoses and dwell in the soil of high-temperature and low-precipitation regions. As climate change expands the fungal geographical range, infections are rising along with the increasing endemic region [5]. There are no preventative vaccines available for humans and therapeutic options are limited. By adopting these computational resources, researchers can now systematically dissect the complex relationships among active enzymes, membrane-bound virulence factors, secreted effectors, and secondary metabolites that work together promote fungal pathogenicity. This integrated computational approach addresses current research bottlenecks and opens new possibilities for structure-based drug design, reverse vaccinology, and systems-level analysis.

## 2. Computational Framework for Fungal Virulence Discovery

**Figure 1:**
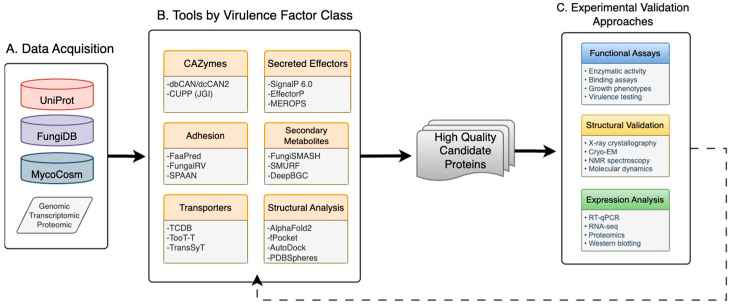
Conceptual computational workflow schematic for virulence factor identification in respiratory dimorphic fungi. This schematic represents the general sequence of research steps and methodological approach common within the field and can be adapted to specific research questions and available resources. The workflow integrates (**A**) data acquisition from public repositories or experiments, (**B**) computational analysis using specialized tools for different virulence factor classes, and (**C**) experimental validation. (**B**) Computational tools organized by virulence factor categories, including CAZymes, adhesins, transporters, secreted effectors, secondary metabolites, and structural analysis tools. (**C**) Experimental validation approaches including functional assays, structural validation methods, and expression analysis techniques. The dotted line depicts how experimental results refine prediction and accuracy through an iterative workflow.

Identifying virulence mechanisms in respiratory dimorphic fungi in silico requires a systematic four-stage workflow (Figure 1). The workflow outlined in Figure 1 is representative of the approaches increasingly utilized in respiratory fungal pathogenesis research to serve as a conceptual road map. The process begins with data acquisition from public repositories such as UniProt, FungiDB, or MycoCosm, or through generation of experimental transcriptomic datasets (Figure 1A) [6,7,8]. Raw genomic or proteomic data is read, trimmed, and aligned prior to beginning this workflow. Protein sequences should be validated for completeness and redundancy to avoid statistical bias, reduce compute time and memory requirements. The next step, selecting computational tools, should not be based solely on availability but on alignment with biological objectives, input data type, prediction quality, interpretability and feasibility for downstream validation (Figure 1B). Tools also differ in the type of input they accept, the databases they draw on, and the extent to which they have been benchmarked against fungal pathogens. Considering the computational cost, interpretability of results, and ease of integration with downstream analyses are equally important. Because prediction algorithms vary in accuracy, precision, and recall, filtering candidates based on confidence metrics or statistical cutoffs helps reduce false positives and focuses attention on biologically meaningful hits. Filtering prediction outputs using statistical metrics reduces downstream screening numbers, removes poor quality candidates and narrows down to biologically relevant factors. This framework provides an iterative process requiring wet-lab validation to confirm prediction accuracy and functional relevance (Figure 1C). The computational saying “garbage in, garbage out” reflects the importance of starting with high-quality input data and tools aligned with research goals increases the likelihood of identifying genuine virulence determinants. The following sections describe commonly used computational approaches, their applications in protein mediated pathogenicity and their potential in advancing *Coccidioides* studies.

## 3. Therapeutic Target Prioritization Framework

To guide experimental efforts, we developed a prioritization framework based on established drug development criteria (Table 1). High-priority targets were defined as those meeting multiple favorable criteria: (a) druggability through defined binding pockets or well-characterized inhibition mechanisms; (b) selectivity via minimal homology to human proteins, reducing off-target effects; (c) essentiality—critical functions in pathogenesis or survival; and (d) precedent from successful targeting in other fungal pathogens or related systems. This classification framework guides experimental validation priorities and informs structure-based drug design efforts (Table 1). Table 1 presents our assessment of therapeutic target potential for each virulence factor class based on established drug development criteria including target accessibility, selectivity, conservation patterns, essential cellular functions and previous antifungal developments. We classify targets as high-priority when representing species-specific adhesins and essential transporters lacking human homologs, while moderate-priority targets such as CAZymes may present challenges due to metabolic redundancy or requirements for selective inhibition strategies.

## 4. *Coccidioides*: A Model Pathogen for Computational Virulence Studies

*C. immitis* and *C. posadasii* are dimorphic fungi with a two-phage lifecycle alternating between saprophytic mycelia in soil and parasitic spherules in mammalian hosts [9]. Arthroconidia are small barrel-shaped yeasts produced by fragmented mycelia that are highly stress-tolerant, thrive in arid, alkaline soils and are easily aerosolized during soil disturbances [9]. Once aerosolized, arthroconidia can be inhaled into terminal bronchioles and alveoli, where they undergo morphogenetic transition into large, multinucleated spherules and ultimately rupture releasing endospores [9,10]. This developmental switch is an essential virulence determinant, as spherule maturation causes remodeling of the fungal cell surface and intracellular proteome [11,12]. Mature spherules evade host immunity through multiple mechanisms. They suppress nitric oxide species in macrophages, and their large multinucleated structure physically impedes phagocytosis by host phagocytes [11,13,14]. This morphological shift is critical for immune evasion because as the chitin- and β-glucan-enriched spherule cell wall is remodeled, immune recognition by host pattern recognition receptors (PRRs) such as Dectin-1 and TLR2 is lost [11,12,15]. Spherules also express stage-specific factors, including the spherule outer wall glycoprotein (SOWgp) and secreted proteases, which contribute to adhesion, tissue invasion, and immune modulation. Transcriptomic and proteomic analyses confirm extensive remodeling of metabolic and cell wall biosynthesis pathways during this phase making spherule-specific factors strong candidates for therapeutic application and relevance in mammalian hosts [12,16,17]. Previous reviews comprehensively detailed *Coccidioides* virulence mechanisms [18,19].

**Figure 2:**
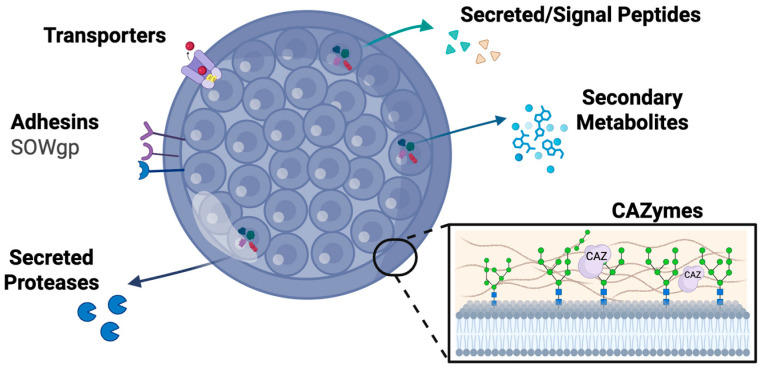
Computational protein targets in *Coccidioides* spherules. Mature *Coccidioides* spherule showing the distribution of major virulence factor classes identified through computational approaches. Surface-associated adhesins, including the spherule outer wall glycoprotein (SOWgp), mediate host cell attachment and colonization [9,10,11]. Membrane-embedded transporters facilitate nutrient acquisition, iron sequestration, and drug efflux [12]. Secreted proteases and signal peptide-containing effectors are released into the host environment to promote tissue invasion and immune modulation [13]. Secondary metabolites produced by biosynthetic gene clusters contribute to host immune suppression and tissue damage [14]. Carbohydrate-active enzymes (CAZymes) in the cell wall (inset) enable morphological transitions and immune evasion through cell wall remodeling [15]. Each protein class represents computationally tractable targets for structure-based drug design, reverse vaccinology, and therapeutic intervention strategies.

The availability of high-quality genomes, population-level comparative data, and stage-specific gene expression profiles positions *Coccidioides* as an ideal system for computational identification of virulence determinants (Figure 2) [16,17,18]. However, our knowledge remains sparse across virulence protein categories and classes. SOWgp represents the most thoroughly characterized *Coccidioides* adhesin, functioning as a key mediator of host cell interaction and virulence. Transporter families make up 13–17% of the proteome, but functional roles for most transporters remain largely unknown [19]. Transcriptomic studies reveal that over 40% of spherule-specific secreted effectors encode proteolytic enzymes, highlighting their potential importance in host–pathogen interaction, yet defined mechanisms are limited [13]. Computational tools can be used to systematically predict candidate virulence factors across adhesins, transporters, secreted effectors, carbohydrate-active enzymes, and secondary metabolites, overcoming limitations of homology-based annotations that miss *Coccidioides*-specific innovations. The following sections detail each virulence class, highlighting how computational predictions accelerate pathogen biology understanding and guide targeted experimental validation.

## 5. Adhesins Mediate Host Attachment and Colonization

Adhesion proteins, which attach to host cells and colonize host tissues, are a well-reported virulence factors in pathogenic fungi [20,21]. The best-characterized adhesion protein in *Coccidioides* is SOWgp, which binds to host extracellular matrix components such as laminin, fibronectin, and collagen [10]. Depleting SOWgp in mouse infections leads to decreased virulence, confirming its essential role in pathogenesis [9]. Because SOWgp is *Coccidioides-*specific, it represents a high-priority target for inhibitors or vaccines as it is less likely to affect commensal fungi or human proteins (Table 1). In *Aspergillus* species, the polysaccharide galactosaminogalactan (GAG) contributes to both host surface attachment and immune evasion by inhibiting neutrophil extracellular traps and activating macrophage inflammasomes [22]. *Candida albicans* uses the agglutinin-like sequence (Als) protein family, especially Als1 and Als3, which act as multifunctional adhesins mediating initial host cell attachment and tissue invasion [23]. Along with Als, other GPI adhesins and cell wall proteins such as secreted aspartyl proteases (Sap), Hsp90, and Hyr/Iff family members critically contribute to fungal host interaction and pathogenesis and are being considered in vaccine development [24].

Predicting fungal adhesion proteins computationally remains challenging due to their sequence diversity and structural complexity [25]. Unlike many other protein families, adhesion proteins lack conserved domains and have high variation across fungal species, making traditional homology-based approaches insufficient [25]. This diversity reflects the evolutionary pressure for fungi to adapt their adhesion strategies to different hosts and environmental conditions. Several bioinformatic tools like FaaPred, FungalRV and SPAAN were developed to identify putative adhesins using machine learning and non-homology-based methods (Table 2). FaaPred is a support vector machine (SVM)-based tool to predict adhesin-like fungal proteins with high specificity [26]. FungalRV, is a user-friendly web-based platform for predicting adhesins from protein sequences using Hidden Markov Model (HMM) machine learning to compare query protein sequences to known adhesions. While initially designed and trained for bacterial species, SPAAN uses a non-homology-based neural network approach to identify putative adhesins across diverse phyla without relying on sequence similarity constraints, which is useful for discovering novel adhesins [26,27,28]. These tools highlight the high sensitivity and specificity across diverse pathogenic fungi achieved by FungaRV. Other predictors like FaaPred may miss key adhesins or yield lower-confidence predictions, emphasizing a need for algorithms capable of handling broad sequence diversity and non-homology among fungal adhesins [25]. Nath A. developed a machine learning framework for fungal adhesin prediction using a dataset of 75 fungal adhesins and 341 non-adhesin sequences with a range of sequence and evolutionary features [29]. SVM ensemble approaches performed best, particularly those with radial basis function (RBF), pearson VII function-based universal kernel (PuK) and polynomial kernels (PolyK), achieving up to 98% validation accuracy and 94.9% cross-validation accuracy. The study found that fungal adhesins often have higher threonine and cysteine content and lower phenylalanine and methionine content, features that help distinguish adhesins from other proteins. Compared to FaaPred, which uses a single SVM and achieves lower accuracy (86–87%) and Matthew’s correlation coefficient (MCC) (0.61), Nath’s ensemble approach demonstrates greater sensitivity in detecting diverse adhesin types, including those lacking conserved domains or present in underrepresented taxa.

There is a strong biological need for new and more advanced tools and models in fungal adhesion prediction. Current approaches are mostly adapted from bacteria because of the limited catalog of fungal adhesins. Fungal adhesins, such as the *Als* family genes*,* contain genomic tandem repeats that contribute to allelic and phenotypic variation allowing attachment to diverse hosts and tissues. These genes are likely to evolve faster than the rest of the genome, accumulate more mutations, and experience recombination events that alter adaptability to changing environments [46]. Existing models often miss newly evolved or highly divergent adhesins because the models are trained on a narrow or static set of known sequences and cannot keep pace with fungal evolution. These challenges emphasize the need for new computational tools to make proteome-scale studies of fungal adhesins more robust [20]. Proteome mining can identify candidate adhesins, but experimental validation through fungal attachment assays and biofilm formation are still required to confirm and characterize their roles [47]. Three-dimensional structural modeling can also validate these predictions; however, adhesins present unique obstacles. Adhesin proteins are often glycosylated, producing heterogeneity that is difficult to capture computationally, and their large, repetitive architectures hinder accurate folding predictions as well as experimental structure determination [20]. Focusing on specific domains, domain-based modeling approaches such as ab initio methods and Rosetta, can help address these challenges [48,49]. For example, ab initio and Rosetta modeling, applied to the tandem repeat domains of *Candida* agglutinin-like sequence (Als) adhesins, reveal compact β-sheet-rich folds with conserved hydrophobic surfaces and structurally stable features, despite glycosylation and sequence repetition [50]. AlphaFold also accurately predicts the structure of individual Ig-like and T domains within Als5, assigning high confidence to folded regions while flagging less ordered repeats as low confidence [50]. Structurally, malleable domains and high-confidence neural network predictions allow for characterization of ligand-binding regions or interaction surfaces even when the full protein is unresolved [49]. Future advances in adhesin prediction could create a curated fungal adhesin training dataset comprising experimentally validated proteins across ample species, weighted toward mammalian pathogens and developing dimorphic fungi-specific models. This resource dataset would account for phase-specific expression patterns and post-translational modifications like O-mannosylation, which are enriched in *Coccidioides* spherule surface proteins [51]. Overcoming these barriers and advancing adhesin prediction resources is important for understanding how pathogenic fungi colonize host tissues and for guiding the development of antifungals and other therapeutic strategies.

## 6. Membrane Transporters in Iron Acquisition and Drug Resistance

Transporters embedded in fungal cell membrane represent high-value targets due to their essential roles in nutrient acquisition, stress resistance and drug efflux [12,52]. As *Coccidioides* spherules mature, membrane-associated proteins interact with host immune cells, acquire nutrients, and resist stress conditions induced by the immune system. Transporters are particularly significant for respiratory pathogens like *Coccidioides*, *Paracoccidioides*, and *Aspergillus*, as they enable survival in the nutrient-scarce environment of the lungs [52].

Genome mining revealed 1288 *C. immitis* and 1235 *C. posadasii* transporter homologs, highlighting the rich transport machinery of *Coccidioides* that likely facilitated its divergence from the typical plant-associated lifestyle of Ascomycota fungi and its adaptation to mammalian host niches [19]. These transporters account for approximately 13–17% of the proteomes and span seven major classes, 25 subclasses, and 269 families, indicating a rich library of substrate translocators essential to nutrient acquisition and stress resistance during infection [19]. This extensive transportome also provides a foundation for developing inhibitors.

The *C. neoformans* transporter Uut1 exemplifies an ideal therapeutic target due to its essential role in polysaccharide capsule synthesis combined with minimal human protein homology. This nucleotide sugar transporter is required for capsule formation, and its disruption leads to enhanced fungal clearance by host phagocytes. Importantly, Uut1 shares less than 12% sequence identity with human proteins, significantly reducing the risk of off-target effects during drug development and making it an attractive candidate for selective anti-*Cryptococcus* therapeutics [53,54].

Beyond nutrient acquisition, transporters provide resistance to host-derived antifungal defenses. Pleiotropic drug resistance (PDR) family transporters like Cdr1p in *C. albicans* mediate multidrug resistance through efflux pumps by actively transporting antifungal compounds out of the fungal cell [55]. *Coccidioides* possesses PDR transporter homologs with *C. neoformans* and multidrug resistant exporters (MDR) with *A. fumigatus* likely contributing to fluconazole resistance in coccidioidal meningitis. The identification and functional annotation of transporter proteins rely on computational tools, though limited studies have used advanced classifiers to detect transporter membrane proteins in human fungal pathogens.

Researchers use FungiDB, a database with ample genetic and protein information for different fungi as a key resource in *Coccidioides* genomic and functional studies [56]. The database includes five genome assemblies for *C. immitis* and ten for *C. posadasii* supporting comparative genomic and transcriptomic characterization across strains and species. Several studies have leveraged this resource to compare genomes of *Coccidioides* non-pathogenic relatives, identify mycelia and spherule differentially expressed genes, and evaluate novel gene-prediction tools with *C. immitis* [18,57,58,59]. Ongoing updates to genome assemblies and associated omics datasets within FungiDB are creating new opportunities for refining computational models, expanding comparative frameworks, and accelerating discovery in *Coccidioides* research [7]. The transporter database (TCDB) is a widely used resource in identifying transporters [32]. Additionally, TooT-T and TransSyT are promising tools in outperforming previous transport protein detection tools (Table 2), which could be adopted in future studies investigating respiratory fungal pathogen nutrient acquisition and secretion mechanisms [33,34]. The *Coccidioides* transportome library is a high priority therapeutic target because of key metabolic functions, has high druggability and the success of transporter inhibitors in other infectious diseases [19] (Table 1). Developing transporter-specific inhibitors opens a new avenue for advancing antifungal therapies. Exploring transporter diversity among fungal species might uncover evolutionary patterns in host–pathogen interactions and niche specialization, supporting antifungals that would target conserved transporters across the diversity of respiratory pathogens.

## 7. Secreted Signal Peptides Modulate Most Immunity and Tissue Invasion

Signal peptides and secreted effector proteins represent another critical class of virulence factors that enable pathogenic fungi to manipulate host immune responses and facilitate tissue invasion and support niche adaptation. These proteins are characterized by N-terminal signal peptides that direct their secretion from fungal cells, where they can interact directly with host systems to promote infection and immune evasion. In *Candida albicans*, signal peptides regulate the secretion of aspartyl proteases that degrade host immune proteins [60]. *Aspergillus fumigatus* produces the cytoplasmic protein AfuCSP1, which contains a signal peptide and interferes with alveolar macrophage recruitment and activation, specifically reducing reactive oxygen species production and blunting inflammatory responses [61].

Recent high-density transcriptomic analyses in *Coccidioides* have revealed the complexity and diversity of secreted effectors during the spherule phase, which is unique to this pathogen and essential for virulence [13,62]. These studies have identified novel spherule-specific secreted factors, including the well-characterized SOWgp and multiple secreted proteases such as serine carboxypeptidases, kexins, and metalloproteases. In research by Homer et at, SignalP was used to filter the transcriptome for proteins likely to be secreted during spherulation [37]. This method revealed a subset of spherule-enriched transcripts encoding potential effectors, including cysteine-rich proteins and multiple secreted proteases.

The significance of these secreted factors extends beyond simple protein degradation. *Coccidioides* secretes the metalloproteinase Mep1, which dampens innate immune recognition during endosporulation by digesting SOWgp, a key cell surface antigen, preventing detection by phagocytes [63,64]. Recent analyses demonstrated that over 40% of the predicted spherule-specific effectors in *Coccidioides* encode proteolytic enzymes, consistent with a protease-driven virulence strategy that is also observed in other pathogenic fungi [13]. Several specialized tools, such as SignalP, recently updated to version 6.0, is the primary tool for identifying proteins likely to be secreted by filtering transcriptomes for signal peptide sequences (Table 2). For functional prediction of effector proteins beyond the presence of a signal peptide, EffectorP applies machine learning to distinguish probable effectors within large gene sets, which is particularly valuable for species like *Coccidioides* where many predicted effectors lack homology to annotated proteins in other systems [36,37]. The MEROPS database is used to classify and annotate proteolytic enzymes. Cross-referencing predicted secreted proteases against MEROPS enables functional inference and comparison of protease family composition across species [38]. Such analyses have revealed convergent evolution among fungal pathogens, including *Coccidioides*, *Aspergillus*, and plant pathogens like *Magnaporthe oryzae*, toward secreted proteases as both effectors and mediators of direct host cell damage. Integrating computational prediction tools (SignalP, EffectorP, MEROPS) with transcriptomic profiling has advanced understanding of secreted effectors in fungal pathogenesis and follow-up experimental validation is essential. In *Coccidioides*, these approaches identify spherule-specific effectors, particularly proteases and glycoproteins, as high priority candidates for studying virulence and antifungal targets because they are accessible extracellularly and can be targeted by small-molecule inhibitors (Table 1).

## 8. Cell Wall Remodeling Enzymes During Immune Evasion

Fungi secrete a wide array of carbohydrate-active enzymes (CAZymes) that enable the breakdown, modification, and utilization of complex carbohydrates from the environment or host. These proteins are essential for survival, nutrition and pathogenic mechanisms, supporting fungi as harmless commensals and disease-causing pathogens [65]. These enzymes show species-specific repertoires that reflect diverse ecological niches, with pathogenic fungi typically possessing more virulence-related enzymes than commensal species and bacteria [66].

CAZymes encompass multiple functional classes, including glycoside hydrolases and glycosyltransferases, which play critical roles in mediating lifecycle transitions and cell wall remodeling. These processes are important in morphological changes, immune evasion, and tissue invasion across diverse hosts [67]. In *Histoplasma capsulatum*, specific CAZymes modify the α-glucan-containing cell wall, reducing recognition by host macrophages and enabling the pathogen to establish infection [68]. Similarly, the β-1,3-glucanase Eng1 protein hydrolyzes β-1,3-glycosyl linkages in fungal cell walls to further evade immune detection [69]. Masking pathogen-associated molecular patterns through CAZyme-mediated cell wall modification is used by numerous pathogenic fungi, including *Candida albicans* and *Aspergillus fumigatus* [70,71].

Despite the importance of CAZymes in fungal virulence and immune evasion, few studies examined their role in *Coccidioides* pathogenesis. Mitchell et al. identified an abundance of CAZymes in *C. posadasii* and *C. immitis*, with homologs to related pathogenic fungal enzymes [15]. Similarly, Mead et al. observed sβ-1,3-glucanases and β-1,6-glucanases upregulation in *Coccidioides* spherules critical for establishing infection in mammalian hosts [16]. CAZymes are often involved in multiple metabolic pathways, likely making it challenging to selectively inhibit theses enzymes without disrupting other beneficial microbiota with similar molecules (Table 1). Beyond morphological transitions, CAZymes in *Coccidioides* may contribute to host–pathogen interactions through additional mechanisms similar to other closely related fungi. In *Candida albicans*, β-glucan masking by exoglucanase Xog1 reduces exposure of β-1,3-glucan on the fungal cell surface, avoiding recognition by immune cells [72]. Xog1 and Eng1 endoglucanase trim exposed β-glucan to modulate fungal interaction with host immune cells and promote immune evasion, especially under host signals like lactate or hypoxia [72,73]. Inactivation of XOG1 impairs β-glucan masking increasing immune recognition, cytokine responses, and overall antifungal immunity. Thus, β-glucan “shaving” is a key fungal strategy to evade host immune defenses, thus restricting T cell activation and differentiation.

Identification and functional annotation of CAZymes across fungal species relies on several computational tools, including dbCAN, dbCAN2, and CUPP, which all integrate with the comprehensive CAZy database, a central resource for carbohydrate-active enzyme classification and functional annotation (Table 2). Besides specialized enzyme databases such as CAZy, the Database of Fungal Virulence Factors (DFVF) catalog characterizes virulence proteins across diverse fungal pathogens [74]. Using DFVF can facilitate highlighting key virulence genes through in-depth analysis in *Coccidioides* and other medically relevant fungi. Future research should leverage these computational resources to uncover *Coccidioides* lineage-specific changes in CAZyme families, particularly those associated with adaptation to increasingly warm environments and mammalian hosts. These studies may reveal both unique adaptive strategies and common cell wall remodeling mechanisms shared among pathogenic fungi and predict how *Coccidioides* may adapt to changing environmental conditions. Specifically, as climate change drives chronic soil warming and alters carbon composition, monitoring CAZyme family expansion and diversification across experimental isolates of *Coccidioides* could provide early warning signs of evolving virulence mechanisms.

## 9. Secondary Metabolites as High Potential Therapeutic Targets

Fungi secondary metabolites are small bioactive molecules that often function as virulence factors and represent promising therapeutic targets. These compounds, including polyketides, non-ribosomal peptides, and terpenes, can modulate host immune responses and contribute to tissue damage. An analysis of the *Aspergillus* secretome highlighted targets for antifungal development [75], while a study of phosphatases in *Cryptococcus neoformans* demonstrated the power of combining computational prediction with experimental validation to uncover novel virulence mechanisms [76].

Biosynthetic gene clusters (BGCs) are groups of co-regulated genes that produce secondary metabolites [14,77]. Riedling et al. applied machine learning to predict the bioactivity of fungal secondary metabolites from BGC data. However, due to the limited number of well-characterized fungal BGCs and their associated metabolites, prediction accuracy was only 51–68%, highlighting the urgent need for larger, curated fungal BGC datasets to improve computational models [78]. While specific secondary metabolites in *Coccidioides* remain largely uncharacterized, genome mining has revealed multiple BGC similarities to known fungal toxin pathways. *Coccidioides* spherule-specific genes, located in isolated regions, exhibit significant gene copy number variations, suggesting rapid evolution [79]. Many of these regions are associated with secondary metabolite biosynthetic pathways, implying that *Coccidioides* may use secondary metabolites to modulate host defenses and promote tissue damage, similar to mycotoxin produced during *Aspergillus* infections [80]. Comparative genomics with closely related dimorphic fungi, such as *Histoplasma* and *Paracoccidioides*, could further pinpoint *Coccidioides* bioactive compounds with pathogenic relevance. In *Histoplasma capsulatum*, the secreted proteins CBP1 and YPS3 induce macrophage apoptosis, facilitating disease establishment and extrapulmonary dissemination [81].

Bioinformatics tools like FungiSMASH, a fungi specific derivative of antiSMASH, and SMURF identify and annotate secondary metabolite BGCs [39,40]. Both are web-based and accessible without extensive computational resources (Table 2). FungiSMASH is more sensitive and accurate, detecting well-defined and partial clusters, making it an ideal tool for studying secondary metabolites in *Coccidioides* [82]. SMURF, while effective, is less sensitive for diverse or fragmented gene clusters. Another deep learning-based tool, DeepBGC, leverages neural networks and predicts BGCs by “learning” from large, curated datasets [41]. Unlike FungiSMASH and SMURF, DeepBGC not only identifies candidate gene clusters but also predicts the bioactivity and provides a confidence value and scoring for its prediction.

A recent addition, TOUCAN, is specifically tailored for fungal BGCs and demonstrates superior performance in identifying and recovering gene clusters in fungal genomes compared to both DeepBGC and FungiSMASH [42]. In benchmark studies, TOUCAN achieved higher precision and F1 scores (up to 0.98), particularly excelling in *Aspergillus* species, although it may require additional boundary filtering for optimal results.

Using TOUCAN on *Coccidioides* genomes may identify uncharacterized BGCs predicted to encode immunosuppressive compounds or new mycotoxins. These predictions could then be cross-referenced with experimentally derived metabolomics data, and with known BGCs in other pathogenic fungi, to pinpoint candidates for functional assays. Secondary metabolites and their biosynthetic pathways represent high-priority therapeutic targets due to their small size, allowing for direct inhibition, and their potential for repurposing existing bioactive compounds (Table 1).

## 10. Structure-Based Drug Design: From Prediction to Therapeutics

Three-dimensional protein structure is an essential part of antifungal drug design by revealing binding sites and conformational changes that might occur during infections. The limited number of experimentally solved structures for *Coccidioides* proteins (only 25 as of August 2025) in the Protein Data Bank is a challenge in *Coccidioides* research. However, AlphaFold2 addresses this gap by providing highly accurate structure predictions for hundreds of fungal proteins [43]. Developing improved medications requires identifying proteins absent, or sufficiently different, in humans to enable selective targeting without adverse effects. The HitList pipeline exemplifies this approach, identifying eight novel protein targets across most WHO-priority fungal threats including Erg8, Ilv3, Ilv5, Rib3, and Rib5 while lacking human homologs [83].

Cell wall synthesis and membrane biosynthesis proteins represent proven drug target classes, while surface adhesion proteins offer vaccine and therapeutic development opportunities. Fungi use β-1,3-glucan synthase to build protective cell walls, an enzyme absent in human cells. The drug caspofungin blocks glucan synthase preventing cell wall synthesis. Cry-EM structures of fungal 1,3-β-glucan synthase identified key residues and drug-binding interfaces of the catalytic subunit (Fks1) revealing cellulose synthase-fold, drug binding and enzymatic mechanisms [84]. Caspofungin exposure significantly alters the structure and composition of the cell walls across different *Candida* species. These structures explain how caspofungin triggers cell wall remodeling, causing wall thickening and polysaccharide changes that affect immune recognition and drug susceptibility [85]. Azoles like fluconazole target lanosterol 14α-demethylase (CYP51) alter enzyme conformation offering opportunities targeting membrane biosynthesis [86,87,88]. Crystal structures of *Candida albicans* CYP51 mutants provide templates for designing next-generation azoles with improved efficacy [88]. Similar structural approaches in *C. neoformans* characterized bifunctional GAR/AIR synthetases, farnesyltransferase, and glucosylceramide synthase, advancing drug discovery through detailed binding mechanism analysis [89,90]. Understanding the detailed architecture of *Coccidioides* adhesins like SOWgp could enable design of vaccines or small-molecule inhibitors that prevent lung epithelial attachment.

Applying a structure-based drug discovery process for *Coccidioides* first requires predicting the fungal protein three-dimensional shapes using advanced computational tools such as AlphaFold2 or homology modeling when experimental data are unavailable [43,91]. Next, computational tools are employed to identify sites on the protein surface where small-molecule compounds may interact. High-priority targets include proteins with well-defined binding pockets, minimal human homology, and essential functions in pathogenesis (Table 1). Tools including fpocket, PocketOptimizer, PDBSpheres, BindWeb and InDeep, each offer distinct algorithms for detecting and characterizing these pockets (Table 2). These methods evaluate shape, size, dynamics, and potential for ligand binding [92,93,94,95,96].

Once binding sites are located, virtual screening and molecular docking tools, such as AutoDock, can rapidly simulate and score numerous compound-protein interactions. predicting optimal binding partners [45]. Mapping known resistance mutations onto protein models visualizes how genetic changes might disrupt or prevent drug binding. This iterative process refines candidate compounds before proceeding to experimental validation, providing a systematic approach particularly valuable for understudied pathogens like *Coccidioides* where experimental structures are scarce.

## 11. Reverse Vaccinology: Computational Approaches to Vaccine Design

Reverse vaccinology, accelerates discovery by focusing on proteins that are exposed on the fungal surface, highly immunogenic, and differ significantly from human proteins minimizing potential side effects [97,98]. This computational approach has been successfully applied across multiple fungal pathogens including *Aspergillus* and other disease related fungi [99,100].

Researchers studying *Paracoccidioides* used NetMHCIIpan-4.0 to identify putative T cell epitopes in fungal peptides that would bind MHCII mouse alleles [101]. Following these predictions, in vitro assays revealed candidate peptides that induce protective immunity. Furthermore, in vivo studies in the *Galleria mellonella* confirmed that three *P. brasiliensis* peptides induce protective immunity in an invertebrate model [102]. These studies exemplify how computer predictions can effectively guide subsequent experiments, bridging the gap between theoretical modeling and practical validation.

In C*occidioides*, protective immunity is primarily mediated by T helper 1 (Th1) and Th17 cells [11,103]. While no FDA-approved vaccine currently exists for human coccidioidomycosis, several candidates (such as Δ*cps*1 live-attenuated, recombinant chimeric polypeptide antigen (rCpa1), and epitope-based formulations) are at various stages of preclinical or clinical development [104,105]. The most advanced candidate to date, Δ*cps*1, is protective in dogs and is currently advancing toward human trials [106,107]. Computational vaccine design is being used to enhance and expedite vaccine design. Hurtgen et al. identified T cell epitopes from three CD4+ T cell reactive proteins (Pep1, Amn1, and Plb) using ProPred to predict promiscuous epitope sequences with 80% probability of binding human MHCII molecules [105]. Protective Th1, and Th17 immune activation predictions were validated with murine ELISPOT assays and by immunizing HLA-DR4 transgenic mice, followed by *Coccidioides posadasii* challenge [105].

T cell prediction tools such as NetMHCIIPan, TepiTool, and ProPred identify potential immune targets by predicting how well protein fragments bind to immune system molecules [108,109,110]. B cell prediction software like BepiPred-3.0, finds targets for antibody responses, while safety assessment programs including AllergenFP and AlgPred 2.0 check for potential allergic reactions [111,112,113]. Immune simulation software such as C-ImmSim models how the entire immune system might respond to a vaccine candidate, providing researchers with detailed predictions before moving to experimental testing [114]. Together, these tools form a streamlined pipeline for epitope selection, enabling efficient candidate prioritization and significantly reducing the time and resources required for initial screening.

## 12. Validation Strategies Bridging Computation and Biology

Experimental validation remains the critical bridge between computational prediction and clinical application, serving as the essential confirmation step for all in silico discoveries (Figure 1C). The validation approach must be tailored to the specific virulence mechanism under investigation and the research objectives, requiring careful consideration of model system limitations and biological relevance. Gene knockout studies using CRISPR-Cas9 or traditional homologous recombination techniques offer direct assessment of target essentiality and are particularly valuable for evaluating transporter proteins and CAZymes involved in cell wall remodeling. These approaches enable researchers to create targeted gene disruptions and assess their impact on virulence, growth, and survival under various stress conditions. In vitro cell culture assays using macrophage or epithelial cell lines serve as intermediate validation steps for adhesion proteins like SOWgp and secreted effectors, allowing researchers to test immune evasion capabilities and host–pathogen interactions in controlled environments. Recombinant protein expression and purification enable detailed biochemical characterization of secreted proteases, secondary metabolite biosynthetic enzymes, and membrane-associated proteins through enzymatic assays and structural studies.

Research on *Coccidioides* is challenging because BSL-3 containment limits laboratory facilities, genetic tools are less developed than in model fungi, and the organism grows slowly [115,116]. These factors make large-scale experimental validation demanding and expensive. As a result, computational prioritization has become not just helpful but essential for guiding research. Applying the framework presented here would allow researchers to focus limited experimental efforts on the most promising targets with therapeutic potential. In vivo validation provides the most physiologically relevant assessment of virulence mechanisms and therapeutic potential, though each model system presents unique advantages and limitations. *Coccidioides* research employs diverse experimental models including traditional murine systems, invertebrate *Galleria mellonella* larvae, naturally infected dogs, non-human primates, and patient studies [117,118,119]. Mouse models remain the gold standard for pulmonary coccidioidomycosis studies, though researchers must carefully interpret results considering that disease progression occurs more rapidly in mice than humans, potentially affecting pharmacokinetic studies and therapeutic evaluation. Invertebrate models like *G. mellonella* offer advantages for high-throughput screening and reduced ethical constraints while providing valuable insights into innate immune interactions.

For example, de Oliveira et al., characterized extracellular proteins using liquid chromatography–mass spectrometry with elevated energy (UPLC–MS^E^) and compare the secretome of two *Paracoccidioides* isolates (*Pb*01 and *Pb*Epm83). They identified 92 secreted proteins with 35 differentially secreted in *Pb*01 and 36 in *Pb*Epm83 and functionally annotated most of these as related to adhesion and virulence processes. Infection assays with macrophages further demonstrated that differences in secreted protein profiles among *Paracoccidioides* isolates [120]. Similarly, Begum et al. computationally characterized CAZyme metabolic pathways in *Candida albicans* using bioinformatics tools like dbCAN2. Follow-up RT-qPCR experiments confirmed elevated expression of genes such as TAZ1, CKI1, SPE3, and FAS1/FAS2, consistent with their predicted enzyme functions [121].

## 13. Computational Challenges and Future Perspectives

Effective use of computational tools in fungal pathogenesis depends on thoughtful selection criteria and interpretation frameworks. Algorithm selection must align with the pathogen’s biology and intended clinical context, as tools optimized for plant or bacterial models can mislead when applied to human fungal pathogens. A pervasive challenge is reliance on non-fungal training data: SPAAN was developed for bacterial adhesins, EffectorP trained on plant pathogen effectors, and several transporter classifiers lack fungal-specific datasets. Bacterial adhesins have pili and autotransporters while fungal adhesins use GPI-anchored glycoproteins; plant pathogen effectors target different immune system mechanisms than mammalian pathogens encounter. Users should mitigate these biases by applying stringent confidence thresholds, requiring multiple lines of computational evidence, and cross-validating with fungal-specific databases (CAZy, FungiDB, DFVF).

Prediction accuracy varies significantly even among similar algorithms; for instance, while both FungalRV and FaaPred use support vector machine approaches, they demonstrate different predictive accuracy for fungal systems. Training set bias, conserved protein sequences, and overreliance on homology all complicate discovery of novel, lineage-specific virulence targets, especially for understudied fungi like *Coccidioides*. Many essential proteins share significant similarities, complicating identification of pathogen-specific regions and often resulting in shorter target sequences that increase off-target risks in antifungal development. To achieve meaningful impact, computational predictions must be translated into experimental validation and clinical action. Three fundamental obstacles hinder progress: (A) Training data scarcity and taxonomic bias limit generalization to understudied dimorphic fungi. Expanding curated, experimentally validated datasets for these pathogens should be a community priority. (B) Accounting for phase-specific biology is essential, as dimorphic fungi express distinct proteomes in environmental and parasitic states. Future tools should explicitly integrate lifecycle-specific transcriptomics to guide appropriate validation approaches. (C) The validation gap is widening as many groups publish tools without experimental follow-up. Practical steps include requiring experimental benchmarking, organizing community validation exercises, and publishing both positive and negative results.

Integrating clinical context such as immune status, prior antifungal use, infection site, and drug penetration can increase translational relevance when ranking target candidates. As experimental datasets grow and algorithms improve, these computational tools will become increasingly powerful for guiding antifungal discovery. Continued collaboration between computational and experimental researchers will be essential for realizing the full potential of these tools and strengthening the public health impact of fungal pathogen research.

## Figures and Tables

**Table 1:** Virulence factor categories in respiratory dimorphic fungi: pathogenic roles, computational identification features, and therapeutic targeting potential.

Virulence Factor Class	Function in Pathogenesis	Computational Signatures	Example Proteins in *Coccidioides*	Conserved Across Species	Therapeutic Target Potential
CAZymes	Cell wall remodeling, immune evasion, morphological transitions	Glycoside hydrolases, Glycosyltransferases, Carbohydrate Transferases, Polysaccharide Lyases families; signal peptides	β-1,3-glucanases, β-1,6-glucanases	High (*Candida, Aspergillus*, *Histoplasma*)	Moderate—metabolic redundancy
Adhesins	Host cell attachment, colonization, biofilm formation	Lack conserved domains; rich in Ser/Thr; repetitive sequences	SOWgp (spherule outer wall glycoprotein)	Low—host-specific adaptations	High—species-specific targets
Transporters	Nutrient acquisition, drug efflux, stress resistance	Transmembrane domains; ABC, MFS families	Sit1-like iron transporters, ABC efflux pumps	High—essential metabolic functions	High—druggable targets
Iron Acquisition	Host iron sequestration, immune suppression	Siderophore biosynthesis clusters; iron-binding domains	Siderophore transporters, iron reductases	High—conserved iron metabolism	High—iron-limiting strategies
Secreted Proteases	Tissue invasion, immune evasion, host protein degradation	Signal peptides; protease domains (M, S, C families)	Mep1 metalloproteinase, serine carboxypeptidases	High—convergent evolution	Moderate—protease inhibition
Secreted Effectors	Host immune modulation, virulence enhancement	Signal peptides; small size; cysteine-rich	Cysteine-rich proteins, secreted hydrolases	Variable—pathogen-specific	High—immunotherapy targets
Secondary Metabolites	Immune suppression, tissue damage, antibiosis	BGC organization; NRPS, Polyketide synthase domains	Putative mycotoxin clusters	Moderate—chemical diversity	High—small molecule inhibition
Membrane Proteins	Stress response, cell wall integrity, morphogenesis	Transmembrane domains; GPI anchors	Cell surface glycoproteins, stress sensors	High—essential cellular functions	High—membrane-accessible targets
Morphogenesis Factors	Yeast-hyphal transitions, spherule development	Stage-specific expression; cytoskeletal interactions	Spherule-specific transcription factors	Moderate—dimorphic fungi	High—morphology disruption

Abbreviations: ABC, ATP-binding cassette; BGC, biosynthetic gene cluster; CAZy, Carbohydrate-Active enzymes database; MFS, major facilitator superfamily.

**Table 2:** Computational tools for fungal virulence factor prediction. This table summarizes the primary bioinformatics tools used to identify different classes of virulence factors, including their methodological approaches, input requirements, key features, and limitations. Tools are organized by virulence factor class to facilitate method selection for specific research applications.

Virulence Factor Class	Tool Name	Method/Algorithm	Input Required	Key Features	Performance Metrics	Limitations	Reference
CAZymes	dbCAN	HMM-based annotation	Protein sequences (FASTA)	Comprehensive CAZy family classification, batch processing	98% accuracy; 2 min per 1000 proteins	Requires manual curation for novel families	[30]
	dbCAN2	Multi-method integration	Protein/genomic sequences	Combines HMM, DIAMOND, Hotpep methods	Sensitivity 95.6%; specificity 97.8%	Computationally intensive	[30]
	CUPP (JGI)	Machine learning pipeline	Assembled genomes	Automated functional annotation	Applied to JGI MycoCosm database	Limited to JGI-hosted genomes	[31]
Adhesins	FaaPred	Support Vector Machine	Protein sequences	High specificity for fungal adhesins	Sensitivity 82.6%; accuracy 86% (fungal dataset)	Limited training dataset	[26]
	FungalRV	Hidden Markov Model	Protein sequences	User-friendly web interface	Sensitivity 82.4%; precision 92.3%; accuracy 99% (fungal dataset)	Moderate sensitivity	[27]
	SPAAN	Neural network	Protein sequences	Non-homology based	89% accuracy (bacteria); 65–75% (fungi)	Originally designed for bacteria	[28]
Transporters	TCDB	Homology-based search	Protein sequences	Comprehensive transporter classification	Contains 20,000+ characterized transporters	Manual annotation required	[32]
	TooT-T	Machine learning	Protein sequences	High accuracy for transport prediction	94% accuracy; 15–20% improvement over BLAST	Limited fungal-specific training	[33]
	TransSyT	Multi-feature analysis	Protein sequences	Outperforms traditional methods	F1-score 0.91; precision 89%	Requires computational expertise	[34]
Secreted Effectors	SignalP 6.0	Deep learning	Protein sequences	High accuracy signal peptide prediction	Precision 94%; recall 91%; <5 min per 10,000 sequences	May miss non-classical secretion	[35]
	EffectorP	Machine learning	Protein sequences	Distinguishes effectors from other secreted proteins	Sensitivity 92%; specificity 88%	Limited training on fungal effectors	[36,37]
	MEROPS	Database search	Protein sequences	Protease classification and annotation	5000+ characterized proteases; E-value < 1 × 10^−5^ threshold	Requires homology to know proteases	[38]
Secondary Metabolites	FungiSMASH	Rule-based + ML	Genomic sequences	Fungi-specific BGC detection	85–95% detection of known BGCs; 20% more sensitive than SMURF	Requires complete genome assemblies	[39]
	SMURF	Rule-based	Genomic sequences	Web-based, user-friendly	Effective for canonical BGC architectures	Less sensitive than FungiSMASH	[40]
	DeepBGC	Deep learning	Genomic sequences	Predicts bioactivity with confidence scores; exploits Pfam domains	BGC detection: 79% precision, 74% recall; bioactivity: 51–68% accuracy	Requires large training datasets	[41]
	TOUCAN	Machine Learning	Genomic sequences	Outperforms FungiSMASH and DeepBGC	Precision 98%; Recall 91%; F1 score 0.98 on *Aspergillus niger* and *A. nidulans*	Possible overprediction of cluster bounds; requires post-process filtering	[42]
Structural Analysis	AlphaFold2	Deep learning	Protein sequences	Highly accurate structure prediction	Median pLDDT > 90 for ordered regions; CASP14 GDT_TS 92.4	Limited to single chain proteins	[43]
	fpocket	Geometric algorithm	Protein structures (PDB)	Druggable pocket identification	94% success rate; 2–5 s per protein	Sensitive to structure quality	[44]
	AutoDock	Molecular docking	Protein structure and ligands	Virtual screening capabilities	RMSD < 2 Å (78% of test cases); 10,000× faster than prior version	Requires expert parameter tuning	[45]

Abbreviations: BGC, biosynthetic gene cluster; BLAST, Basic Local Alignment Search Tool; CASP, Critical Assessment of protein Structure Prediction; CAZy, Carbohydrate-Active enZYmes database; DIAMOND, Double Index Alignment of Next-generation sequencing Data; FASTA, Fast-All, a text-based format for representing nucleotide or peptide sequences; GDT_TS, Global Distance Test Total Score; HMM, hidden Markov Model; Hotpep, Homology and Peptide pattern recognition; JGI, Joint Genome Institute; ML, machine learning; PDB, Protein Data Bank; Pfam, Protein families database; pLDDT, predicted Local Distance Difference Test; RMSD, root-mean-square deviation; TCDB, Transporter Classification Database.

## Data Availability

No new data were created or analyzed in this study. Data sharing is not applicable to this article.
